# Editorial: Perceptual Linguistic Salience: Modeling Causes and Consequences

**DOI:** 10.3389/fpsyg.2017.00411

**Published:** 2017-03-22

**Authors:** Alice Blumenthal-Dramé, Adriana Hanulíková, Bernd Kortmann

**Affiliations:** ^1^Department of English, University of FreiburgFreiburg, Germany; ^2^Freiburg Institute for Advanced Studies, University of FreiburgFreiburg, Germany; ^3^Department of German, University of FreiburgFreiburg, Germany

**Keywords:** prediction, language learning, morphology, salience, surprisal, social markers, dialects, language variation and change

Recent years have seen an upsurge of interest in the notion of salience in linguistics and related disciplines. The attention literature distinguishes two broad types of perceptual salience (Summerfield and Egner, 2009; Awh et al., 2012). First, a stimulus can be salient—i.e., foremost in one's mind—because it is cognitively preactivated. This type of salience, sometimes referred to as top-down salience, may occur if a stimulus is expected because it is part of a cognitive routine, if it has recently been mentioned, or due to current intentions of the perceiver. Research on salience as a semantic-pragmatic phenomenon has shown that top-down salience can account for systematic preferences in the interpretation of figurative utterances, pronominal antecedents, implicatures, and discursive links (Geeraerts, 2000; Giora, 2003; Chiarcos et al., 2011; Jaszczolt and Allan, 2011).

While in top-down salience, perceivers endogenously direct their attention to a certain stimulus, in the second type of salience, bottom-up salience, it is the stimulus itself which attracts attention. In prototypical cases of bottom-up salience, the stimulus stands out because it is incongruous with a given ground by virtue of intrinsic physical characteristics. But a stimulus may also cause surprise by virtue of deviating from a cognitive ground, e.g., when violating social or probabilistic expectations (Clark, 2013). This has prompted researchers to examine the relationship between expectations and the perceptual salience of linguistic stimuli in new ways (Hanulíková et al., 2012; Rácz, 2012; Hanulíková and Carreiras, 2015; Blumenthal-Dramé, 2016a,b; Roller, 2016; Blumenthal-Dramé et al., 2017), and inspired us to organize a workshop devoted to this particular area.

In October 2014, the Freiburg Institute of Advanced Studies (FRIAS) hosted the workshop “Perceptual linguistic salience: Modeling causes and consequences”, organized by the editors of this volume. Bringing together researchers from psycholinguistics, sociolinguistics, neurolinguistics, and cognitive linguistics, the workshop sought to explore the notion of perceptual salience and its explanatory potential for the domains of language processing, variation, and change. Several questions arising from the stimulating discussions were listed in the call for papers for this Research Topic and included the following:

- Which cognitive processes underlie the differential treatment of salient vs. non-salient linguistic percepts?
- How can these processes be accommodated within psycholinguistic models?
- How can the perceptual salience of linguistic forms and variants be operationalized?
- To what extent is salience an intrinsic feature of linguistic forms (e.g., dialectal variants), and to what extent does it result from contextual factors or prior experience with language?

This volume features nine contributions including five original research articles, one review, and three commentaries that addressed the above questions in very interesting ways. Several contributions discuss which factors or prior experience with language underlie the differential treatment of salient linguistic percepts, and how can they be operationalized and modeled. Jaeger and Weatherholtz argue that sociolinguistic salience can be quantified using computational psycholinguistics. A distinction is made between the initial salience of a novel variant and the cumulative product of experienced exposures to a variant. A variant's salience may be predicted based on its surprisal and frequency. In support of this view, Schmid and Günther propose a unified framework of salience which aims at reconciling seemingly contradictory uses of this notion in the literature: cues are either categorized as salient because they confirm expectations, or because they violate them. Zarcone et al. suggest that an articulated model of salience should take into account attention, affect, and predictability at different levels of processing, and that these dimensions and their interactions can be straightforwardly accommodated within the Predictive Coding framework. Finally, Giraudo and Del Maso present a critical review of so-called decompositional accounts of morphological processing. They argue that the salience of morphemes cannot be reduced to formal factors, and that semantic factors and relationships between holistically represented complex words should also be integrated into models of morphological processing.

Several contributions address the hypothesis that salient items might function as cognitive reference points that structure and give access to certain cognitive domains (e.g., sociolinguistic stereotypes), thereby influencing the perception and categorization of less salient items of the same domain (Rosch, 1975; Langacker, 1993; Hanulíková and Weber, 2012). On the basis of recent theories of enregisterment and exemplar processing, Jensen investigates percepts resulting from sociolinguistic or socio-cognitive salience, more exactly the salience of various morphosyntactic forms in vernacular Tyneside (Northeast England). This study brings to the fore the role of place as strongly shaping both a community's and an individual's linguistic identity and self-representation. Llamas et al. present metrics for determining the relative salience of phonetic variables in the Scottish-English border zone. This paper substantiates the fact that the choice of features which ultimately become sociolinguistically salient is largely arbitrary. What matters is sufficient agreement among the members of the relevant speech community as to which structural features are considered to function as signals of group membership. Using eye-tracking, Grohe and Weber show for regional dialects of German that salience clearly has an effect on native accent adaptation, but only if objective criteria for salience apply.

The notion of perceptual salience is inextricably linked to issues concerning language acquisition. Cintrón-Valentín and Ellis examine effects of physical salience and attentional biases in the visual and auditory modalities in second language acquisition. Chinese and English native speakers were trained on Latin tense morphology under different types of explicit form-focused instructions, some of which successfully increased learners' attention to less salient morphological features. Rácz et al. use artificial language learning and show that the social-cognitive salience of non-linguistic contexts influences learning of morphological features. Learning is easier with a coherent and interpretable social context (such as gender of the speaker) as opposed to accidental links between the speaker and the construction (such as front-facing vs. side-facing).

Taken together, the papers featured in this volume contribute to our understanding of how the perceptual salience of linguistic forms and variants can be theoretically framed and methodologically operationalized in different areas of linguistic processing.

## Author contributions

All authors listed, have made substantial, direct and intellectual contribution to the work, and approved it for publication.

## Funding

Funding for the workshop was provided by the Freiburg Institute for Advanced Studies (FRIAS) at Albert-Ludwigs University in Freiburg, Germany.

### Conflict of interest statement

The authors declare that the research was conducted in the absence of any commercial or financial relationships that could be construed as a potential conflict of interest.
